# Adsorption Performance of Activated-Carbon-Loaded Nonwoven Filters Used in Filtering Facepiece Respirators

**DOI:** 10.3390/ijerph16111973

**Published:** 2019-06-04

**Authors:** Małgorzata Okrasa, Jörn Hitz, Aleksandra Nowak, Agnieszka Brochocka, Christoph Thelen, Zbigniew Walczak

**Affiliations:** 1 Department of Personal Protective Equipment, Central Institute for Labour Protection, National Research Institute, Wierzbowa 48, 90-133 Łódź, Poland; alnow@ciop.lodz.pl (A.N.); agbro@ciop.lodz.pl (A.B.); 2 Sub-Division 3.3 “Personal protective equipment against Chemical and Biological Substances”, Institute for Occupational Safety and Health of German Social Accident Insurance (IFA), Alte Heerstraße 111, 53757 Sankt Augustin, Germany; joern.hitz@dguv.de (J.H.); christoph.thelen@dguv.de (C.T.); 3 Department of Theoretical Physics, University of Lodz, Pomorska 149/153, 90-236 Łódź, Poland; z.walczak@merlin.phys.uni.lodz.pl

**Keywords:** activated-carbon-loaded nonwovens, respiratory protection, filtering facepiece respirators, anti-odour properties, breakthrough time

## Abstract

Filtering nonwovens loaded with activated carbon are among the most popular materials used in the construction of filtering facepiece respirators (FFRs) with anti-odour properties that can be used for respiratory protection at workplaces where the occupational exposure limits of harmful substances are not exceeded. Such FFRs, in addition to a polymer filter material of varying effectiveness, also contain a layer of activated-carbon-loaded nonwoven filter, which limits the quantity of chemical compounds entering the breathing zone. The aim of this work was to analyse the influence of challenge concentration (20–120 ppm), relative humidity (2–70%), flow rate (20–55 L/min), and flow pattern (steady-state and pulsating) on the breakthrough of polymer/carbon nonwovens. A commercial activated-carbon-loaded nonwoven filter was used in this study. Its morphology and textural parameters were determined using optical microscopy, image processing, and nitrogen adsorption/desorption measurements at 77 K. Breakthrough experiments were carried out using cyclohexane vapours to assess adsorption characteristics of polymer/carbon media. The results showed that the breakthrough times decreased with increasing challenge concentration (up to 30%), relative humidity (up to 73%), and flow rate (up to 72%). The pulsating flow pattern was found to be more favourable in terms of odour reduction efficiency (up to 30%). The results indicate that all of these factors should be considered during selection and performance assessment of respirators used for odour relief.

## 1. Introduction

According to the generally accepted definition, odorous compounds are substances that at a sufficient concentration may elicit a response in the olfactory and trigeminal system in the nose [1]. Regardless of its origin (natural or man-made), odour pollution has been associated with adverse health effects in humans (mainly self-reported), including irritation of eyes and upper respiratory tract, headaches, nausea, drowsiness, and sore throat [2,3,4]. Prolonged odour exposure has also been connected with behavioural symptoms like stress, sleep disturbance, mood swings, and depression [4,5]. Due to the growing awareness of the general public concerning the influence of air quality on health and wellbeing, these issues have become the subject of regulatory interest world-wide [6]. Simultaneously, extensive research on deodorization techniques for industrial and agricultural facilities [7,8,9] and methods for determining the effectiveness of such techniques have been carried out [10,11,12].

Little attention was paid to the matter of exposure to odorous compounds in a work environment in the past. In situations where the occupational exposure limits (OEL) of harmful substances are exceeded at a workplace and there is no possibility of preventing occupational risk by eliminating it at source or minimizing it by organizational solutions or technical protection measures, it is necessary to use appropriate personal protective equipment [13]. In particular, protection against harmful vapours and gases can be achieved by using appropriate respiratory protective devices, such as a facepiece (halfmask/full-face mask) with gas filters or combined filters containing adsorbent materials with a high surface area such as activated carbon [14]. The testing methods for such devices and the influence of different operational conditions influencing their performance are widely discussed in the literature and well established in international standards [12,14,15,16].

However, when the OEL values are not exceeded, disposable filtering facepiece respirators (FFRs) with anti-odour properties are often in use. In addition to polymer filtering materials of varying effectiveness, they also contain a layer (or several layers) of activated-carbon-loaded nonwoven, which adsorbs some of the volatile chemical compounds that otherwise would enter the breathing zone. Contrary to typical gas filters or combined filters, the performance assessment methods of anti-odour FFRs have not been clearly established in the literature. Only limited research is available on the adsorption characteristics or capabilities of such FFRs [17]. Because the basic principle of operation of anti-odour FFRs is similar to gas filters (adsorption on activated carbon), it is quite straightforward to use a similar performance assessment method. However, the nature of the factors determining the adsorption kinetics on thin carbon-loaded nonwovens (usually thinner than 1 cm) may be different than for densely-packed carbon beds of higher thickness (up to 10 cm) that are found in gas filters. This is mainly due to the specific phenomena that occur during the two-way flow of the above-mentioned substances through a flat and loosely-packed bed that is a mixture of polymer fibres and grains or granules of activated carbon adsorbent.

Water vapour is another factor that profoundly influences the performance of activated carbon beds found in gas filters [18,19,20,21], which may be of significance when considering breakthrough times of polymer/carbon beds used in FFRs for odour relief. Studies using standard FFRs showed that relative humidity under the facepiece can reach over 90% within a few minutes of its use [22]. Warm and humid air exhaled through the thin polymer/carbon bed can easily interact with activated carbon, leading to very poor adsorption conditions (high humidity and heating of the carbon). Those conditions persist in the material over the whole breathing cycle, affecting the adsorption also during inhalation. Moreover, the exhaled air can cause the migration of moisture into the facepiece structure and its accumulation between the hydrophobic fibres of the nonwoven material [23]. This effect is negligible in the case of typical gas filters used in combination with half-masks or full-face masks because they are usually equipped with exhalation valves that prevent the humid exhaled air from entering the gas filter. However, it may be significant in the case of anti-odour FFRs in which the flow is bidirectional, even if an exhalation valve is installed.

Another of the factors that should be considered when analysing the process of uptake of volatile compounds by these types of polymer/carbon beds is the composition of the absorbed mixture. In general, carbon adsorbents have a greater affinity for less volatile substances, but there is no unambiguous relationship between the single characteristics of an individual chemical compound and the capacity of the sorption bed [24]. In the case of individual chemical compounds, the breakthrough time of the activated carbon bed, under given ambient conditions, is usually described using the original or modified Wheeler‒Jonas equation [25,26,27,28]. But when there is a mixture of various compounds in the air, as in the case of odour exposure, the problem of determination of breakthrough time becomes much more complex [29,30].

For densely-packed carbon beds, the breakthrough time was also found to be inversely proportional to the volumetric flow rate of air/adsorbate mixture for a given organic vapour, concentration, and relative humidity [31]. In this case the flow pattern (steady-state or pulsating) had no significant influence on the breakthrough time even for high work rates and humidity conditions. However, for carbon beds of low thickness (1.0–3.5 cm) the dependence of the breakthrough time on the flow pattern has been confirmed and attributed to the influence of flow rate on the mass transfer from the bulk gas phase to the surface of the adsorbent particles [32]. It can therefore be assumed that the flow pattern will also have considerable influence on the breakthrough time of nonwoven filters loaded with activated carbon. However, hitherto, no studies on the issue have been conducted.

The present study addresses some of the aspects connected with the adsorption characteristics of FFRs with anti-odour properties. Namely, the influence of material morphology, challenge concentration, relative humidity, flow rate, and flow pattern on the breakthrough of polymer/carbon media is considered.

## 2. Materials and Methods

### 2.1. Activated-Carbon-Loaded Nonwoven Filters

A commercially available activated-carbon-loaded nonwoven filter (AC-NW composite, type RK-270/C, Remark-Kayser, Batorowo, Poland) typically used in the construction of FFRs with anti-odour properties was used in this study. It was manufactured from poly-(ethylene terephthalate) (PET) by needle-punching (160 g/m^2^) and impregnated with 110 g/m^2^ of powdered activated carbon.

### 2.2. Morphology of Activated-Carbon-Loaded Nonwoven Filters

The basis weight of AC-NW media was determined according to methodology described in EN 29073-1:1992 standard [33] using R160P laboratory balance (Sartorius GMBH, Göttingen, Germany). Their thickness was established according to ISO 9073-2:1995 standard [34] using the J-40-T digital material thickness gauge (CheckLine, Bad Bentheim, Germany). The pressure drop was measured according to EN 13274-3:2001 standard [35] using breathing resistance measuring apparatus comprising a blower/sucker system connected via flowmeter to the filter holder and a manometer connected upstream of the sample measuring the pressure drop relative to atmosphere at 30 and 95 L/min air flow rates. Samples (*N* = 21 ÷ 76) for all of the measurements were cut out randomly from different parts of AC-NW media.

**Figure 1:**
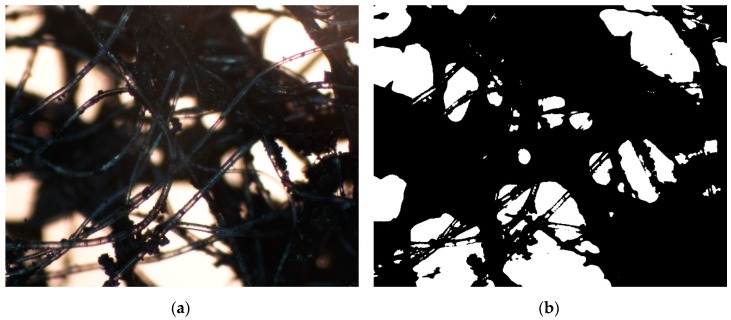
Raw optical microscope image (**a**) and grayscale representation (**b**) of activated-carbon-loaded nonwoven (AC-NW) sample.

Additionally, an Axiotech 100 HD optical microscope (Carl Zeiss CMP GmbH, Göttingen, Germany) equipped with a Nikon DS-Fi1 digital camera and Nikon DS-U2 controller (Nikon Corporation, Tokyo, Japan) was used to evaluate fibre diameter distribution and optical density of AC-NW media. Micrographs taken at magnification of 250x were generated using NIS-Elements BR™ software (Laboratory Imaging Ltd., Prague, Czech Republic) and then processed with the software package Mathematica 9 (Wolfram Research, Inc., Champaign, IL, USA). In particular, the area of the regions which had a darker shade (areas covered by the AC-NW media) compared to the rest of the image was calculated numerically in the following way. First, we used the routine *MorphologicalBinarize* that converts multichannel and colour images into grayscale images and then produces images in which every pixel has value 0 (white pixel) or 1 (black pixel) (Figure 1). Next, we applied the routine *DominantColors* that, for a given grayscale image, determines the fraction of the whole grayscale image covered by clusters of black pixels. This method allowed us to roughly assess the uniformity of AC agglomeration in AC-NW composite without considering the heterogeneity of the base NW nonwoven because transparent PET fibres were omitted by the *MorphologicalBinarize* routine.

The uniformity of the parameters of interest was assessed based on the coefficient of variance (%) and index of dispersion I defined as the ratio between the observed variance and the mean value. I < 1 indicated spatially uniform distribution, I = 1 random distribution, and I > 1 clumped distribution [36].

### 2.3. Textural Properties of Activated-Carbon-Loaded Nonwoven Filters

The adsorption/desorption isotherms of N_2_ at 77 K were measured using an Autosorb iQ analyser (Quantachrome Instruments, Boynton Beach, FL, USA) to determine the textural properties of AC-NW composite. Moreover, activated carbon (AC) extracted from the AC-NW composite was tested to examine the influence of incorporation on those properties. Prior to the adsorption/desorption measurements, each AC-NW sample was fragmented and homogenised. Next, samples weighting 1 g were prepared, placed in bulb cells, and subjected to a two-stage outgassing process in conditions compliant with the recommendations of the National Institute of Standards and Technology (1st stage: 50 °C, 90 min; 2nd stage 120 °C, 240 min) [37] to remove any contaminants already adsorbed on the samples. Adsorption/desorption measurements were carried out at 64 measuring points (P/P_0_ from 10^−7^ to 0.995) and desorption measurements at 40 measuring points (P/P_0_ from 0.995 to 0.025).

The test results were analysed using ASiQwin V. 3.01 software (Quantachrome Instruments, Boynton Beach, FL, USA). The specific surface area was calculated using the multipoint BET method for 6 points at P/P_0_ ranging from 0.01 to 0.15. The volume and size distribution of micropores were determined using the QSDFT (quenched solid density functional theory) model based on a slit-pore model (pore diameter < 2 nm) and a cylindrical pore model (pore diameter > 2 nm).

### 2.4. Adsorption Characteristics of Activated-Carbon-Loaded Nonwoven Filters

**Figure 2:**
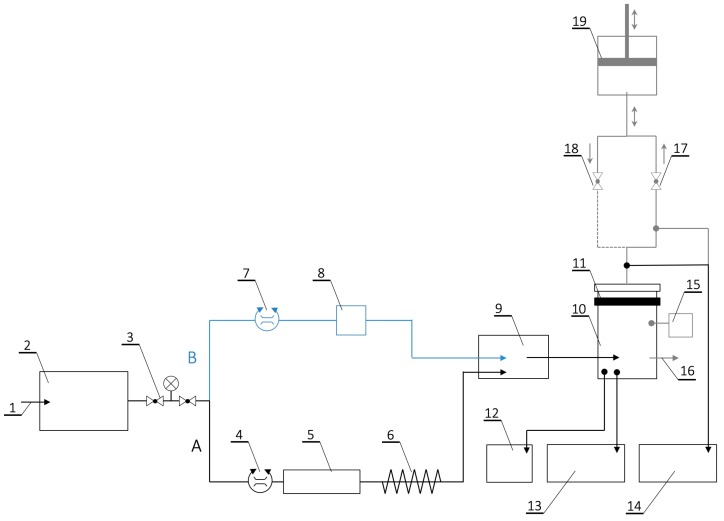
Diagram of breakthrough testing stand: 1—compressed air; 2—dust filter; 3—pressure regulator; 4, 7—mass flow controllers; 5—humidifier; 6—heating/cooling system; 8—evaporator of the test substance; 9—mixing chamber; 10—test chamber; 11—test specimen; 12—integrated temperature and humidity sensor; 13, 14—gas detectors; 15—pressure sensor; 16—exhaust; 17, 18—control valves; 19—breathing machine. Elements 15–19 were used only during pulsating flow tests.

Breakthrough experiments were carried out using cyclohexane vapours as a challenge substance at a test stand shown in Figure 2. Cyclohexane was chosen to perform the experiments due to the fact that it is the representative of the protection group A (organic gases) named in the test standard EN 14387:2004+A1:2008 [16]. Furthermore, the test setup presented in Figure 2 was optimized for the use of cyclohexane as a test substance due to that circumstance. Cyclohexane is a good representative of this group of substances because it is non-polar and unreactive and therefore should be bound to the carbon surface mainly by physical adsorption. Moreover, it is one of compounds that is used in substantial amounts in industrial processes, thus it is often present in the work environment.

During the test, dry compressed air was delivered to the system through a particulate filter equipped with a pressure reducer allowing an adjustment in the range of 0–8 bar. It was then divided into two lines (Figure 2): line A that was used for temperature and relative humidity regulation and line B that was used to adjust the concentration of the test substance in the test chamber. Air at a given temperature and relative humidity from line A was mixed with challenge vapours from line B in a mixing chamber and then directed to the test chamber with the sample (13 × 13 cm) locked in a pneumatic sample holder with a diameter of 11.3 cm (surface area of 100 cm^2^). Additionally, a breathing machine connected to the outlet of the test chamber was used during pulsating flow tests. The breathing machine was set at 25 cycles/min and 2 L/stroke, which corresponded to 25 inhalations and 25 exhalations per minute with a respiratory volume of 2 L. The airflow pattern of the breathing machine was sinusoidal. The challenge concentration during pulsating flow tests was compensated to ensure that the overall load was standardized to the steady-state flow conditions. The challenge vapour concentration upstream (C_in_) and downstream (C_out_) of the sample was recorded using X-am 7000 multi-gas meters (Dräger, Lübeck, Germany) and NGA 2000 MLT 3.1 (Fisher-Rosemount, Wesling, Germany). The temperature and relative humidity in the test chamber were monitored using the VelociCalc® Multi-Function Meter 9545 (TSI, Shoreview, MN, USA). The experiments were performed in four variants, each assessing the influence of a different factor (i.e., challenge concentration, relative humidity, challenge flow rate, challenge flow pattern) on the adsorption characteristics of AC-NW composite. Specific conditions at which the experiments were performed are shown in Table 1.

The measurements were carried out until the concentration of the test substance behind the sample reached the challenge concentration (i.e., the concentration upstream of the sample). Each experiment was carried out in *n* = 5÷8 independent repetitions. Breakthrough times of 50-percent and 100-percent were determined from the breakthrough curves and corrected using the following equation:

(1) $${\mathrm{tb}}_{i\left(\mathrm{corr}\right)}=\frac{{\mathrm{tb}}_{i}\cdot C_{\mathrm{in}\left(\mathrm{measured}\right)}}{C_{\mathrm{in}}},$$

where: tb_i(corr)_—corrected breakthrough time [min], tb_i_—measured breakthrough time [min], i—breakthrough percent, C_in (measured)_—measured concentration [ppm], C_in_—nominal challenge concentration [ppm].

For breakthrough concentration equal to test concentration, the sorption capacity C [g] for studied nonwovens was calculated according to the following equation:

(2) $$C=V\cdot C_{\mathrm{in}}\cdot {\mathrm{tb}}_{100\left(\mathrm{corr}\right)},$$

where: V—challenge flow rate [L/min], C_in_—challenge concentration [mg/m^3^], tb_100 (corr)_ —corrected breakthrough time [min].

### 2.5. Statistical Analysis

Statistical analyses were performed using STATISTICA 13.1 software (Statsoft, Tulsa, OK, USA). Descriptive statistics for all variables of interest were calculated. One-way analysis of variance (ANOVA) at the significance level 0.05 was performed to identify statistical differences between breakthrough times determined for each of the test conditions. When statistical differences were detected (*p* < 0.05), mean values were compared using Tukey’s post hoc procedure at the significance level 0.05.

## 3. Results and Discussion

### 3.1. Morphology of Activated-Carbon-Loaded Nonwoven Filters

**Figure 3:**
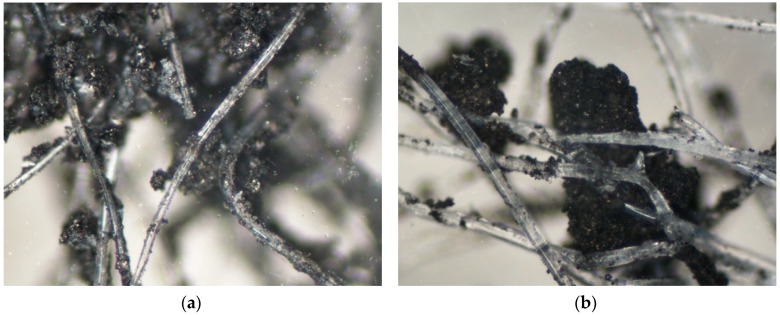
Optical microscope images of AC-NW composite at (**a**) 250x and (**b**) 500x magnification.

The morphology of the nonwoven is shown in optical microscope images (Figure 3).

Considerable accumulation of agglomerated powdered carbon material in the spaces between the tangled polymer fibres was observed. Agglomerates of sizes not exceeding the fibre diameter were present in smaller quantities. They were attached to elementary fibres but not embedded in their structure, which is important because the deposition of carbon adsorbent in the nonwoven structure can significantly impact the sorption properties of the material. This is due to the fact that the presence of binding substances or embedding carbon granules in polymer material may lead to micropore blocking, limiting the resulting capacity of carbon material [38].

The basis weight of AC-NW media ranged between 250 and 321 g/m^2^. The index of dispersion was higher than 1 (Table 2), which indicated that the spatial distribution of mass was aggregated. With such non-uniformity, the adsorption properties between different areas of AC-NW media can differ significantly due to the differences in available adsorption sites. At the same time, the thickness of the nonwoven was quite stable, ranging between 2.1 and 2.7 mm, with low a dispersion index, indicating uniformity.

The AC-NW media had very low pressure drops (Table 2), which can be associated with low breathing resistance when used in the construction of FFRs. According to the EN 149:2001+A1:2009 standard, the breathing resistance of the FFR should not exceed 60–100 Pa at the flow rate of 30 L/min and 210–300 Pa at the flow rate of 95 L/min depending on the protection class [39]. The pressure drop of AC-NW composite constituted less than 1% of the threshold values for both air flow rates, thus the increase of breathing resistance posed by this nonwoven in FFRs would be negligible. Low values of pressure drop along with the coefficients of variance at the level of 6% and 9% indicate that the non-uniformities in terms of this parameter should not affect the sorption characteristics of the AC-NW samples cut from various parts of the material.

Fibre diameters of the AC-NW media were typical of nonwovens obtained via needle-punching ranging from 13 to 30 µm. This parameter affects mainly the filtration of particles and should not be important in the case of sorption properties. However, the impregnation with activated carbon could, to some extent, depend on the fibre diameter as more carbon particles would be trapped in the structure of the nonwoven in the areas where the thinner fibre agglomeration was greater.

Optical densities of the nonwoven varied between 56% and 98%, which additionally confirms that the carbon material was non-uniformly distributed in the polymer structure, i.e., areas with high and low agglomeration of activated carbon were clearly visible. All irregularities in distribution could influence the uptake of volatile chemical compounds from the stream of flowing air.

### 3.2. Textural Properties of Activated-Carbon-Loaded Nonwoven Filters

**Figure 4:**
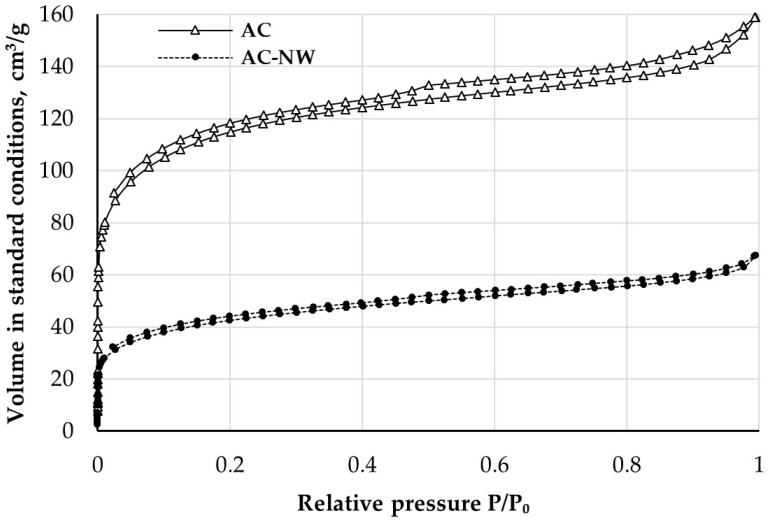
Nitrogen adsorption-desorption isotherms (77 K) of AC and AC-NW composite.

The nitrogen adsorption (lower branch)/desorption (upper branch) isotherms of activated carbon and activated-carbon-loaded nonwoven filter are shown in Figure 4.

Isotherms of activated carbon and activated-carbon-loaded nonwoven were similar to the theoretical type I(b) curve characteristic for microporous materials having pore size distributions over a broad range including wide micropores (0.7–2.0 nm) and narrow mesopores (>2.5 nm) [40]. Its typical feature is the long plateau extending over a very wide range of relative pressures (P/P_0_). In this case, instead of a plateau, a slow increase of the adsorbed volume (as for type II isotherms) was observed, which can be due to nitrogen physiosorption on nonporous polymer fibres or the adhesive used to incorporate the carbon in the nonwoven structure [41]. A rapid increase in the adsorbed nitrogen volume in the low P/P_0_ range indicates the presence of highly developed porous structures in the tested samples.

Textural properties of AC and AC-NW composite calculated using BET and QSDFT models are shown in Table 3.

**Figure 5:**
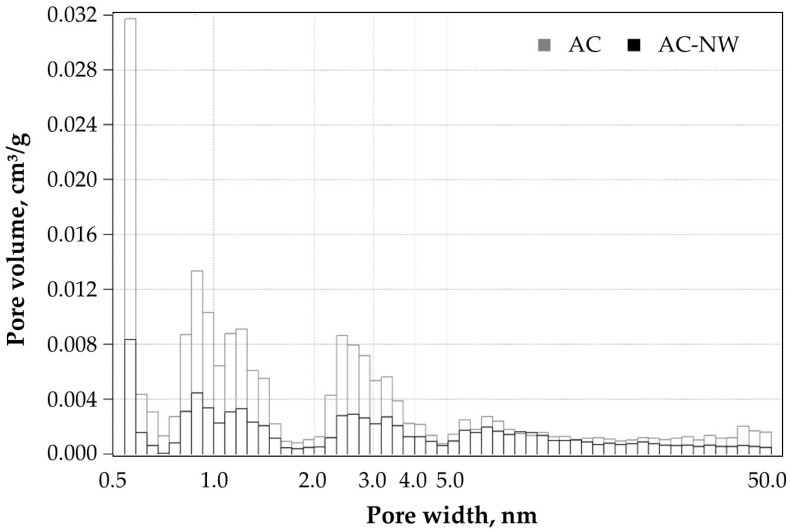
Pore size distribution of AC and AC-NW composite based on QSDFT model.

Good fit of experimental data to the theoretical models was observed (correlation coefficient close to 1 for BET and low fitting error value for QSDFT) (Table 3). At the same time the specific surface area of AC-NW composite was approx. 35% of the specific surface area of the extracted AC, which is due to the high content of non-porous polymer fibres in the tested samples [14]. Lower textural parameters obtained for AC-NW media could have also resulted from blocking of adsorbent pores by the adhesives used in the nonwoven impregnation process [38]. Pore size distribution of AC and AC-NW composite (Figure 5) indicated that the size of most of the pores ranged from 0.5 to 2.0 nm with two peaks at approx. 0.6 and 1.0 nm. Mesopores with dimensions ranging from 2.0 to 50.0 nm were also observed, which might have contributed to the presence of a small hysteresis loop (Figure 4) generally associated with metastability of the adsorbed multilayer, network effects, or pore blocking [37]. Macropores were not observed in either of the samples.

### 3.3. Adsorption Characteristics of Activated-Carbon-Loaded Nonwoven Filters

#### 3.3.1. Influence of Challenge Concentration

**Figure 6:**
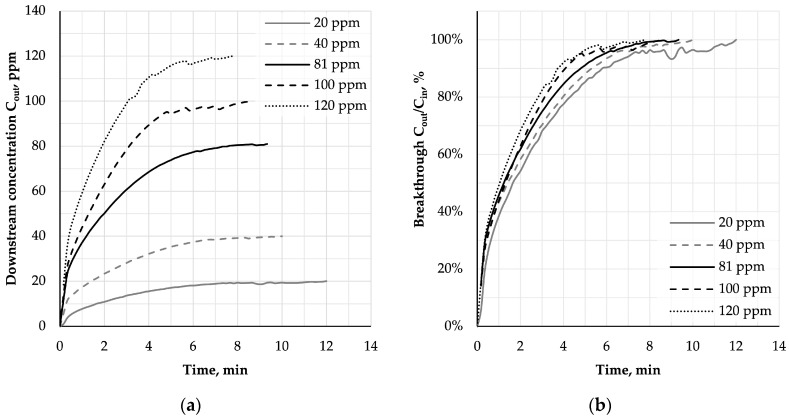
Downstream concentration (**a**) and breakthrough percentages (**b**) for AC-NW media in time.

In Figure 6a the averaged concentrations of cyclohexane downstream of the AC-NW sample obtained for upstream concentration in the range of 20-120 ppm at a steady-state flow of V = 30 L/min, T = 22 ± 3 °C and RH = 70 ± 3% (see Table 1) were plotted versus time. Corresponding breakthrough percentages of cyclohexane vapours through AC-NW media calculated as a ratio of downstream concentration C_out_ to upstream concentration C_in_ (%) are shown in Figure 6b.

In contrast to densely packed carbon beds found in gas filters (depth greater than 2 cm), an initial breakthrough (from 0% to 20%) occurred at the very beginning of each experiment (within the first 30 s), which resulted from the flow of the challenge mixture through the areas of the nonwoven with lower aggregation of AC confirmed by basis weight uniformity analysis. After that, an increase in breakthrough percentage with the increasing upstream concentration of the adsorbate was observed, which is consistent with the results obtained for densely packed carbon beds [42].

**Figure 7:**
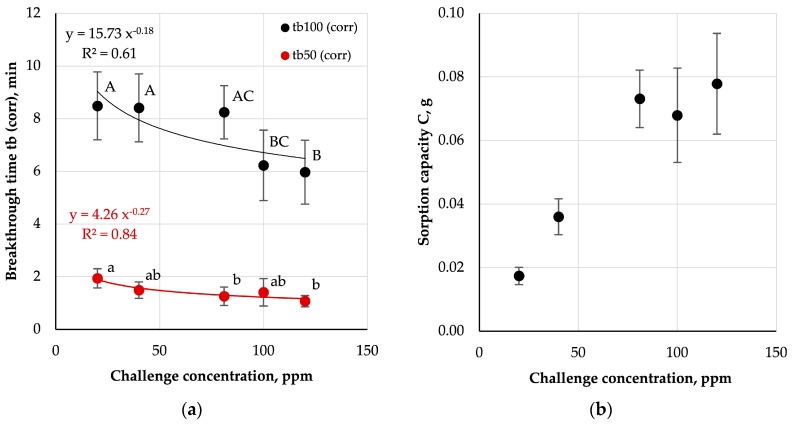
Influence of challenge concentration on (**a**) corrected breakthrough time and (**b**) sorption capacity; A–C, a, b—statistically significant differences between mean values are marked with different letters; ANOVA, *p* < 0.05; Tukey’s test, *p* < 0.05.

Adsorption characteristics of AC-NW composite determined based on the breakthrough curves are shown in Figure 7 along with the results of the statistical analysis.

The 100-percent breakthrough times for AC-NW composite decreased with increasing concentrations (tb_100(corr)_ ranged from 8.5 ± 1.3 min to 6.0 ± 1.2 min). Statistical analysis indicates the existence of some statistically significant differences between averages obtained for various test concentrations (*p* < 0.05, Figure 7). In the case of 50-percent breakthrough times, a more stable diminishing trend was observed with increasing concentration (tb_50(corr)_ ranged from 1.9 ± 0.3 min to 1.1 ± 0.2 min). The sorption capacity increased, with rising challenge concentrations, for the entire range of concentrations except for a minute fall at 100 ppm.

The results of the analyses are largely in agreement with previously published data. Nelson and Harder postulated that the breakthrough time of an activated carbon bed, under given ambient conditions, is proportional to the appropriate power of absorbed substance concentration [43]. Van Osdell et al. confirmed that this relationship can be used to predict breakthrough times of densely packed carbon beds (depth 2.54 cm) for low challenge concentrations performing measurements at high concentrations [43]. The power relationship between breakthrough time and concentration does not constitute a good fit for tb_100(corr)_ time (R^2^ = 0.61). A better fit of the results to the theoretical curve was found for tb_50(corr)_ time (R^2^ = 0.84). These differences may be due to the non-uniformity of AC concentration in AC-NW nonwoven compared to densely packed carbon beds and the very low bed thickness. In the worst case scenario, the difference in carbon content between two 100 cm^2^ samples could be as high as 0.28 g, which would correspond to approx. 115 m^2^/g in available adsorption sites.

#### 3.3.2. Influence of Humidity

**Figure 8:**
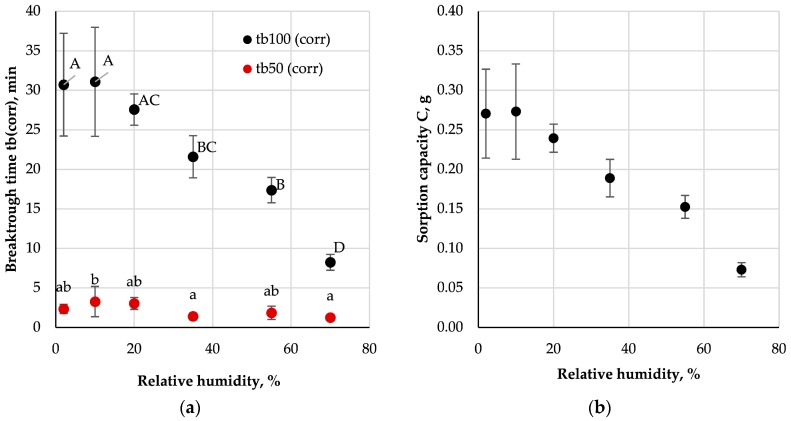
Influence of relative humidity on (**a**) corrected breakthrough time and (**b**) sorption capacity; A-D, a, b—statistically significant differences between mean values are marked with different letters; ANOVA, *p* < 0.05; Tukey’s test, *p* < 0.05.

Breakthrough times and sorption capacities in relation to the relative humidity of the challenge mixture are shown in Figure 8.

The results indicate a significant decrease of 100-percent breakthrough time with an increase in the relative humidity of the challenge mixture (from approx. 30 min for 2% and 10% to 8.2 ± 1.0 min for 70%). In the case of 50-percent breakthrough, no statistically significant differences between times obtained at different relative humidity conditions were found (values ranged from 3.3 ± 1.9 min for 10% to 1.3 ± 0.4 min for 70%). At the same time, a significant reduction in the sorption capacity of the studied beds was observed (from 0.27 ± 0.06 g for 2% and 10% to 0.07 ± 0.01 g for 70%).

The literature describes a number of models characterising the water adsorption process on the surface of carbon materials [44,45,46,47]. In most cases, activated carbon, upon contact, adsorbs water vapour from the challenge stream, thus reducing its capacity to adsorb non-polar organic vapours. The research on respiratory protective equipment indicates that the reduction of sorption capacity of organic vapours with increasing humidity might be much stronger at low challenge concentrations [18,19]. This may have significant implications in the case of carbon-loaded nonwoven filters found in FFRs with anti-odour properties, which can be used up to OEL concentrations.

#### 3.3.3. Influence of Flow Rate

**Figure 9:**
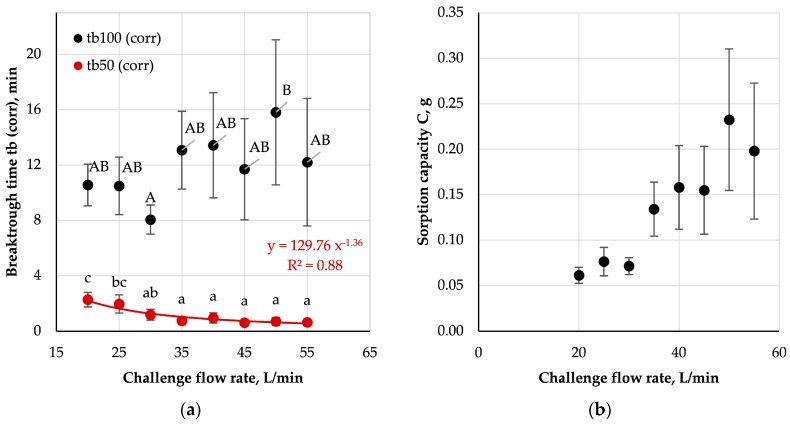
Influence of challenge flow rate on (**a**) corrected breakthrough time and (**b**) sorption capacity; A, B, a-c—statistically significant differences between mean values are marked with different letters; ANOVA, *p* < 0.05; Tukey’s test, *p* < 0.05.

Breakthrough times and sorption capacities as a function of challenge flow rate are shown in Figure 9.

For tb_100(corr)_ no statistically significant decrease was observed with increasing flow rate. On the other hand, for tb_50(corr)_ such a relationship was statistically significant only for a flow rate range of 20–35 L/min. Subsequent semi-constant values of tb_50(corr)_ for the challenge flow rates ranging from 35-55 L/min might have resulted from negligible differences in the adsorption rates in the initial phase of each experiment. For low flow rates, the increase in the test substance concentration downstream of the sample was less rapid than at higher flow rates, which was due to the fact that at low flow rates, the time of interaction of a given volume of the challenge mixture with the polymer/carbon bed was relatively long. This allowed more of the test substance to be absorbed from the challenge mixture resulting in a gradual increase of downstream concentration. With the increase of the flow rate, the time of interaction of the same volume of the challenge mixture with the bed shortened causing a more rapid increase of downstream concentration.

Published research on the dependence of the breakthrough time on the challenge flow rate mainly concerns densely-packed carbon beds found in gas filters. In their case, an inversely proportional relation between the breakthrough time and the flow rate is fulfilled in the conditions of constant concentration and humidity [31]. In the case of carbon-loaded nonwoven filters the relation between those two variables might be even stronger (Figure 9). This means that the differences in breakthrough times for different work intensities will be more clearly visible and should be considered during assessment of the effectiveness of FFRs with anti-odour properties.

The sorption capacity increased with growing challenge flow rate over the entire flow range. This result is consistent with the theoretical predictions, according to which, for a constant concentration of the test substance, the capacity should increase proportionally to the product of the flow rate and breakthrough time.

#### 3.3.4. Influence of Flow Pattern

**Figure 10:**
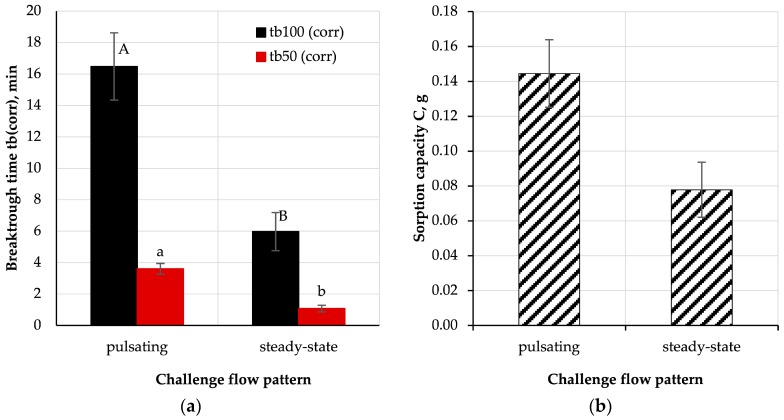
Influence of challenge flow pattern on (**a**) corrected breakthrough time and (**b**) sorption capacity; A, B, a, b—statistically significant differences between mean values are marked with different letters; t-test, *p* < 0.05.

In addition to the challenge flow, the absorption process was also affected by the challenge flow pattern. Figure 10 presents the results of breakthrough experiments performed for steady-state and pulsating flow.

The breakthrough times obtained for steady-state flow were nearly three times shorter than for pulsating flow (*p* < 0.05), which resulted from a shorter interaction time between the challenge stream and activated-carbon-loaded nonwoven filter (only in inhalation phase). It cannot be ruled out that the observed phenomena were also influenced by the sinusoidal changes in the flow rate of the challenge mixture through the sample and the removal of the adsorbate from the polymer/carbon material by the exhaled air. This possibility was previously indicated in the literature in the context of densely-packed activated carbon beds [32,48], however, to fully confirm this effect for carbon-loaded nonwoven filters, desorption experiments should be conducted at various testing conditions. The adverse effect of humidity, originating from the humidifying system located in the exhalation line of breathing machine, on the breakthrough time and sorption capacity was smaller than the effect of the pulsating flow. Hence, the reduction of the breakthrough time due to the accumulation of moisture in the structure of AC-NW composite was not observed. This result is interesting and requires further investigation, with a broader range of testing conditions, as it might have vast implications in terms of the performance of FFRs with anti-odour properties.

## 4. Conclusions

Microscopic analysis has allowed the identification of morphological features that may influence the adsorption characteristics of carbon-loaded nonwoven filters. It was found that the homogeneity of carbon in the polymer fibre structure may have significantly affected those properties. Nitrogen adsorption/desorption measurements allowed the determination of textural properties of the polymer/carbon media. The obtained results indicated the presence of a highly developed porous structure with a minor contribution of mesopores. The breakthrough times decreased with increasing concentration. With the increase in the relative humidity of the test mixture, a significant shortening (even by 30% compared to the experiments performed in dry conditions) of the breakthrough times for the tested beds was observed. The relationship between the breakthrough time and the challenge flow rate fitted a power trend line and was stronger than similar relations found in the literature for densely-packed carbon beds. This may have a direct impact on estimating protection time for respiratory protective equipment containing such materials during work. The test mixture flow mode also had a significant influence on adsorption characteristics. The shortening of the interaction time of the challenge mixture with the polymer/carbon bed was a dominant factor responsible for extending the breakthrough time. However, in this case, the influence of increased humidity of exhaled air, sinusoidal changes in flow, and the phenomena of test substance removal through the exhaled air on the sorption process cannot be ruled out. Although, in general, it is difficult to establish the relationship between odour and concentration of a substance due to interpersonal differences regarding odour thresholds, the proposed approach extends the existing knowledge in the area of performance assessment of FFRs containing activated-carbon-loaded nonwoven filters. Regardless of the limitations, the study results show clearly which of the external factors need to be considered while assessing the efficiency of such respiratory protective devices, which is valuable for health and safety professionals in terms of the evaluation and selection of respirators, as well as establishing times that they can be safely used by workers. Future research in this field should be aimed at developing objective methods for the performance assessment of FFRs with anti-odour properties that would consider inter-individual differences between individuals regarding odour thresholds.

## Figures and Tables

**Table 1:** Testing conditions.

Variable	Range of the Variable	Test Conditions
Challenge concentration C_in_ [ppm]	{20, 40, 80, 100, 120}	Challenge flow rate (steady-state): V = 30 L/min Temperature: T = 22 ± 3 °C Relative humidity: RH = 70 ± 3%
Relative humidity RH [%]	{2, 10, 20, 35, 55, 70}	Challenge concentration: C_in_ = 81 ± 3 ppm Challenge flow rate (steady-state): V = 30 L/min Temperature: T = 22 ± 3 °C
Challenge flow rate (steady-state) V [L/min]	{20, 25, 30, 35, 40, 45, 50, 55}	Challenge concentration: C_in_ = 81 ± 3 ppm Temperature: T = 22 ± 3 °C Relative humidity: RH = 70 ± 3%
Challenge flow pattern F	{steady-state, pulsating}	Challenge concentration: C_in_ = 81 ± 3 ppm Temperature: T = 22 ± 3 °C Relative humidity: RH = 70 ± 3%

**Table 2:** Morphological parameters of AC-NW composite.

Parameter	Basis Weight, g/m^2^	Thickness, mm	Pressure Drop, Pa		Fibre Diameter, µm	Optical Density, %
			at 30 L/min	at 95 L/min		
*N*	76	61	21	21	96	66
Mean value	275.64	2.35	1.43	5.48	22.75	81.82
Standard deviation	17.55	0.11	0.13	0.32	4.63	9.09
Coefficient of variance	6%	4%	9%	6%	20%	11%
Index of dispersion	1.12	0.00	0.01	0.02	0.94	1.01

**Table 3:** Textural properties of AC and AC-NW composite.

Calculation Method	Parameter	AC	AC-NW
BET	Surface area, m^2^/g	419.4	153.2
QSDFT	Fitting error, %	0.15	0.16
	Pore width, nm	0.85	0.85
	Average pore volume, cm^3^/g	0.22	0.09
	Surface area, m^2^/g	418.9	144.4

BET—Brunauer–Emmett–Teller; QSDFT—Quenched Solid Density Functional Theory
